# Rapid resolution of concussion related symptoms in an 11‐month‐old male with integrative medicine assessment and treatment: Case report

**DOI:** 10.1002/ccr3.1993

**Published:** 2019-01-15

**Authors:** Myung Kyu Chung, Patrick J. LaRiccia

**Affiliations:** ^1^ Cooper Medical School of Rowan University Moorestown New Jersey; ^2^ Center for Clinical Epidemiology and Biostatistics Perelman School of Medicine University of Pennsylvania Philadelphia Pennsylvania

**Keywords:** autonomic response testing, concussion(s), injections, local anesthetic(s), neural therapy

## Abstract

Neural therapy may have promise as a helpful, fast acting, and safe method of treating concussion symptoms.

## 1 INTRODUCTION

An 11‐month‐old male with a history of concussion related symptoms: increased crying, increased irritability, and visual motor changes for 14 days after head trauma presented to the office. There was total resolution of symptoms during the office visit after neural therapy treatment following assessment which included autonomic response testing.

Sequelae of concussions can result in long term morbidity.^1^, ^2^ The leading cause of concussion in children <5 years of age is falls.^3^, ^4^ Concussions without loss of consciousness can be difficult to diagnose especially in children <3 years old.^4^ Irritability and visual system changes can present in the context of concussion.^1^, ^2^, ^4^, ^5^ The autonomic nervous system and neuronal depolarization appear to be involved in concussion pathophysiology.^4^, ^6^

We present a case of an 11‐month‐old male with a clinical diagnosis of concussion related symptoms who had a rapid dramatic response after integrative medicine assessment and treatment. The assessment technique of autonomic response testing (ART) was followed by the integrative medicine treatment method known as neural therapy (NT). Both ART and NT are described below. The mother of the patient has provided signed written consent for publication of this report.

### 1.1 Autonomic response testing (ART)

Autonomic response testing is a form of applied kinesiology (AK). AK is a form of manual muscle testing in which an interpretation is made regarding the response (weakness, no change, or strengthening) of a muscle to manual testing. The interpretation informs the assessment of the patient and the prediction of positive, negative, or neutral responses to therapies.^7^, ^8^ ART was developed by Dietrich Klinghardt, MD, PhD, and Louisa Williams, DC, ND.^9^ Many chiropractors and holistic/integrative medicine practitioners incorporate some form of AK within their practices. There are various forms of AK of which ART is one. AK originated with George Goodhardt, Jr, DC.^10^

The various versions of AK use different muscle testing protocols and thus may provide conflicting results with each other. The various methods of AK were developed by their originators to increase the sensitivity and accuracy of testing. Our recent study indicated good accuracy when ART assessment of allergy was compared to an Immunoglobulin E blood test.^11^

### 1.2 Neural therapy (NT)

Neural therapy (NT)^12^ is a method that uses injections of local anesthetic into trigger points, scars, peripheral nerves, autonomic ganglia, tendon and ligament insertions, the epidural space, and tissues. The determination of injection site(s) depends on the findings of a conventional medical evaluation in the context of referred pain, dermatomes, regional influence of autonomic ganglia, and identified interference fields (IFs). IFs can be generated by any damaged tissue via chronic stimulation of afferent neurons in the autonomic nervous system resulting in chronic autonomic reflex activity such as nausea, vomiting, and pain. Scars are often sources of interference fields. NT was developed in Germany and the former USSR in the first half of the 1900s. It is commonly used in German‐and Spanish‐speaking countries, but poorly known in the English‐speaking world. It has been used for the treatment of multiple medical conditions, injuries, and pain.^12^, ^13^, ^14^, ^15^, ^16^, ^17^, ^18^, ^19^, ^20^, ^21^, ^22^, ^23^, ^24^, ^25^, ^26^, ^27^, ^28^, ^29^, ^30^

The IF concept was developed to explain some of the of the NT effects. Scars, fractures, dental lesions can create ANS disturbances that can affect other organs. Signals from IFs can provide nonanatomically connected interference with body areas and organs remote from the damaged tissue Approved producing the IF. Over the past 90 years, reports have documented clinical experience suggesting that injecting an IF such as a scar or cutaneous segment overlying the location of the IF can resolve pain, organ dysfunction, or other symptoms near or at a distance from the IF.^12^, ^13^, ^14^, ^15^, ^16^, ^17^, ^18^, ^19^, ^20^, ^21^, ^22^, ^23^, ^24^, ^25^, ^26^, ^27^, ^28^, ^29^, ^30^

Local anesthetic injections, like procaine, have the capacity to repolarize the nerve cell membrane resulting in restoration of organ function.^12^ Nerve cell polarization is posited to be the crucial NT mechanism^12^ in contrast to endorphin release in acupuncture.

Segmental therapy involves the creation of subcutaneous blebs (also known as quaddels) in the skin several inches apart over a specific area in order to remove an interference field over a given organ.^13^

## 2 CASE PRESENTATION

### 2.1 Visit 1

An 11‐month‐old Caucasian male was brought to the office by his mother. On the day of the visit while at daycare, the child rolled off the changing table and landed on his head on a nonpadded floor. There was no loss of consciousness, no seizure like activity, or any vomiting. The patient's mother who was not present at the day care center when the event occurred. She was called to the daycare center. The mother took the child to the child's pediatrician who found no major issue and advised head trauma precautions. Immediately after the visit to the pediatrician, the patient was then taken to our office. The child's past medical history was noncontributory. He had normal neurological milestones prior to this visit. *Physical examination* revealed a small, reddened area just above his right orbital region. ART testing of the head was negative. He was assessed as having a head contusion.

### 2.2 Plan

His mother was given standard head trauma precautions and no specific brain injury treatments. No analgesics were prescribed, recommended or provided by the child's mother.

### 2.3 Visit 2, 14 days later

The patient's mother reported that since the fall the child was more irritable and had a change in personality. He cried more often and didn't seem to make full, alert eye contact with her. *Physical examination* revealed a child who appeared disengaged with his external environment and irritable. The rest of the standard medical physical exam was unremarkable. ART using the surrogate format was positive over the frontal region of the patient's head. The clinical impression was that the patient's irritability, change in personality, increased crying, and lack of alert eye contact were the result of a concussion that occurred from the patients fall that occurred 14 days earlier.

### 2.4 Treatment

Based on MKC's prior training and experience in NT a NT treatment was performed. The child was given eight ≤0.5 mL subcutaneous injections of 0.5% preservative free procaine in a circumferential manner around the level of his forehead in a headband‐like distribution spaced 3 inches apart. After completion of the injections, the mother exclaimed, “He's back!” After the procedure it became even more apparent how his previous behavior was abnormal when his now normal behavior emerged. He had a bright‐eyed look about him, laughed, and was fully engaged in his environment. No analgesics were prescribed, recommended or provided by the child's mother.

*Follow‐up with the patient 4 weeks* later indicated no post head trauma sequelae. An interview with the patient's mother *8 years later* indicated no head trauma sequelae. The patient's mother called the results of the treatment a “miracle” and related that the day after the treatment the child's day care teacher remarked that the child was back to normal.

The patient was seen originally in May of 2010 and the mother interviewed regarding the head trauma visits in June 2018. The purpose of the 8 year follow‐up interview was to ascertain the duration of the treatment effect and whether or not any late head trauma sequelae had developed. All assessments were clinical in nature as this report is based on a retrospective chart review not a prospective study with standardized questionnaires and protocols.

## 3 DISCUSSION

An 11‐month‐old male child with concussion related symptoms of increased crying, irritability, personality change, and visual motor change dramatically became symptom free right after an in office NT treatment. There were no side effects and the patient has remained free of concussion related symptoms for the past 8 years. The ART testing was consistent with the presence of an IF in the head which is consistent with current thinking about the pathophysiology of concussions.^4^, ^6^ The ART testing was consistent with a brain localization IF as a result of the patient's head trauma with consequent increased irritability, crying, and lack of full, alert eye contact.

Recently, the CDC released guidelines informed by multiple authorities regarding the diagnosis and management of concussion (mild traumatic brain injury‐mTBI).^31^, ^32^ Our presented case occurred 10 years ago. The diagnosis of our presented case was based on the clinical history and physical diagnosis. Current recommendations are the use of published checklists and decision rules. Current management of cases without intracranial brain injury include observation, gradual re‐introduction of activity, education, counseling, nonopioid analgesia for headache, and referral for problems such as vestibule‐ocular dysfunction. Routine CT and MRI scanning is not recommended. It is often ignored that evidence based medicine includes patient preferences and clinical expertise.^33^, ^34^, ^35^ In this case, presentation the patient's mother's preference was to seek the expert opinion of an integrative medicine practitioner after consulting with her child's pediatrician. On the second office visit regarding the patient's fall, NT was performed based on the experience of the clinician and at the preference of the patient's mother. The result was immediate and permanent resolution of the signs and symptoms of irritability, increased crying, personality change, and nonalert eye contact. Since the patient was only 11 months old the presence or absence of headache which can occur following concussions could not be ascertained. Further, costly time consuming referrals to specialists was avoided as was consideration of analgesic administration.

The limitations of this report are that it is retrospective and is one single case. Retrospective case reports sit at the bottom of the evidence hierarchy however they are valuable in pointing out new directions of attention looking for better therapies. Single case reports can be confounded by concomitant interventions along with the possibilities of spontaneous remission, placebo response, and regression to the mean. The patient had received no concomitant interventions, and it is unlikely that full spontaneous remission or complete regression to the mean occurred suddenly and exactly at the time of the injections. It is also unlikely that the 11‐month‐old child had such great expectation or faith in the treatment that a placebo response was the reason for improvement. Further research evaluation is justified by our clinical experience. Hopefully, this case report will stimulate the research community to investigate NT for treatment of postconcussion symptoms with more sophisticated research designs and on a larger scale. We are open Approved to collaboration. However, we caution the reader that this is not an instructional report. Use of the methods in this report requires the acquisition of the proper training provided by the appropriate professional organizations and institutions.

### 3.1 Summary

NT may be helpful in the treatment of concussion related symptoms. Further research is warranted.

## ETHICAL APPROVAL

Our study does not require ethical approval. The patient's mother gave written permission for publication.

## CONFLICT OF INTEREST

None declared.

## AUTHOR CONTRIBUTIONS

MKC: performed the clinical assessment and clinical procedures, wrote the case presentation, and critically reviewed the manuscript. PJL: performed the literature search, wrote the introduction and discussion sections, interviewed the patient's mother, and clarified sections of the case presentation.
